
JOURNAL INFORMATION
==============================
NLM Title Abbreviation: Innov Aging
Journal ID: Innov Aging
NLM Journal ID: 101703706
Journal ID: innovateage

Innovation in Aging

EISSN: 2399-5300
Publisher: Oxford University Press

ARTICLE INFORMATION
==============================
PMCID: PMC10736567
DOI: 10.1093/geroni/igad104.2939
Article ID: igad104.2939
Article version: 1
Subjects: Abstracts, Session 7830 (Poster), Cognition and Cognitive Impairment, AcademicSubjects/SOC02600

CHRONIC VASCULAR COMORBIDITIES WITH SUBJECTIVE COGNITIVE DECLINE IN ADULTS WITH CANCER

Komalasari Renata Penn State Nursing, State College, Pennsylvania, United States

Ryant Adhe S Dirgahayu School of Health Sciences, Samarinda, Kalimantan Timur, Indonesia

Publication date: 2023 Dec
Electronic publication date: 2023 Dec 21
Volume: 7
Issue: Suppl 1
Issue title: Program Abstracts from The GSA 2023 Annual Scientific Meeting, “Building Bridges > Catalyzing Research > Empowering All Ages”
First page: 914
Last page: 914
Copyright: © The Author(s) 2023. Published by Oxford University Press on behalf of The Gerontological Society of America.
Copyright year: 2023
License: This is an Open Access article distributed under the terms of the Creative Commons Attribution License (https://creativecommons.org/licenses/by/4.0/), which permits unrestricted reuse, distribution, and reproduction in any medium, provided the original work is properly cited.
License URL: https://creativecommons.org/licenses/by/4.0/

Page count: 1

==============================

Abstract citation ID: igad104.2939

### CHRONIC VASCULAR COMORBIDITIES WITH SUBJECTIVE COGNITIVE DECLINE IN ADULTS WITH CANCER

Renata Komalasari[1], and Adhe S. Ryant[2], *1. Penn State Nursing, State* College, *Pennsylvania, United States*, *2. Dirgahayu School of Health Sciences, Samarinda, Kalimantan Timur, Indonesia*

People with cancer self-reported memory problems nearly twice as likely than their counterparts, increasing difficulties in daily performances. Despite expected relationships between chronic vascular comorbidities and subjective cognitive decline (SCD), studies are yet to map the likely chain mediation of psychological distress and functional difficulties in the relationship of these variables. This study aimed at examining the association between chronic vascular comorbidities and SCD by psychological distress and functional difficulty mediation in adults with cancers. We utilized the 2019 wave of the Behavioral Risk Factor Surveillance System dataset on 263 adults aged 45 to 80 with cancer (41.1% males and 58.9% females). Chain mediation analysis using 5000 bootstrap samples showed that chronic vascular comorbidities significantly and directly affect SCD. Psychological distress and functional difficulties serially mediated the relationship (indirect effect = .14, 95% CI .05 to .26), with a 26.9% overall mediation effect. Psychological distress alone did not mediate the relationship between chronic vascular comorbidities and SCD (indirect effect = .09, 95% CI -.08 to .27). Functional difficulties mediated the relationship between chronic vascular comorbidities and SCD (indirect effect = .29, 95% CI .05 to .57). These findings suggest that greater chronic vascular comorbidities are associated with a higher sense of SCD through psychological distress which may be magnified by greater functional difficulties. These findings extend the prediction of the chronic stress theory that emotional reactivity to daily functional stressors may exacerbate SCD among adult cancer survivors. These findings may inform person-centric intervention for adults with cancer to self-monitor functional difficulties.
